# Technology-assisted intervention for parents of adolescents in residential substance use treatment: protocol of an open trial and pilot randomized trial

**DOI:** 10.1186/s13722-016-0067-4

**Published:** 2017-01-03

**Authors:** Sara J. Becker, Lynn Hernandez, Anthony Spirito, Selby Conrad

**Affiliations:** 1 Center for Alcohol and Addictions Studies, Brown University School of Public Health, 121 South Main Street, Box G-121-5, Providence, RI 02912 USA; 2 Department of Psychiatry and Human Behavior, Brown University Medical School, Box G-BH, Providence, RI 02912 USA

**Keywords:** Adolescent, Substance use disorder, Residential, Technology

## Abstract

**Background:**

Adolescents in residential substance use disorder (SUD) treatment have poor outcomes post-discharge, with follow-up studies suggesting that most adolescents relapse within 90 days. Parenting practices directly influence adolescent SUD outcomes, but parents of adolescents with SUDs are difficult to engage in traditional behavioral treatments. The current study adapts and evaluates a technology-assisted intervention for parents of adolescents in residential SUD treatment. Based on pilot qualitative data with parents, adolescents, and residential staff, we augment an existing computerized intervention (Parenting Wisely; PW) with four in-person coaching sessions, personalized text messages, and an expert-moderated online parent message board. We hypothesize that parents will find enhanced PW (PW+) both feasible and acceptable, and that adolescents whose parents receive PW+ will have better post-discharge outcomes than adolescents who receive standard care (SC) only.

**Methods/design:**

A two phase approach is used to adapt and evaluate PW+. Phase 1 consists of an open trial with 10 parents of adolescents (age 12–17) in residential SUD treatment. Post-discharge qualitative and quantitative data from parents and adolescents will support PW+ refinement. Phase 2 is a randomized pilot trial with 60 parents testing the effectiveness of adding PW+ to SC. Adolescents and parents will complete assessments at baseline, 6-, 12-, and 24-weeks post-discharge. Primary outcomes will be measures of feasibility and acceptability. Secondary outcomes will include adolescent substance use, truancy, high-risk sexual behavior, and criminal involvement. Two parenting processes (monitoring and communication) are examined as potential mediators of change.

**Discussion:**

This study will adapt and evaluate a technology-assisted parenting intervention as a means of improving adolescent outcomes following residential SUD treatment. Results have the potential to advance the field by: addressing a high-risk population, improving parental engagement; targeting parenting practices (putative mediators of change) that have been linked to adolescent outcomes; and developing a highly disseminable approach.

## Background

According to the most recent data from the National Survey of Substance Abuse Treatment Services, there are 3450 residential substance use treatment facilities in the United States and 10.3% of all adolescents who seek treatment for a substance use disorder (SUD) will receive treatment in this setting [1]. Adolescents with SUDs who require a residential level of care typically have the most severe symptoms and the highest rates of psychological, behavioral, legal, motivational, environmental, and vocational problems [2, 3]. Adolescents in residential SUD treatment are also at extremely high risk of relapse, with follow-up studies suggesting that 60% of adolescents discharged from residential SUD facilities will relapse within 90 days [4–6]. Despite such high relapse rates, the majority of adolescents in residential treatment either do not link to or only participate minimally in continuing care after discharge [7, 8]. In recognition of these challenges, a review of the continuing care literature [9] advised treatment developers to supplement or replace traditional clinic-based approaches with new service delivery approaches that are of lower burden and greater convenience, and that facilitate sustained contact with the patient for an extended period of time. Consistent with these recommendations, this study aims to adapt and evaluate a flexible, low cost, low intensity treatment model for parents preparing for their teen’s discharge from residential treatment.

### Rationale for targeting parents

Parents have been established as a critical influence on adolescents’ initiation and maintenance of substance use [10], as well as their substance use outcomes and likelihood of relapse following treatment [11]. Two parenting processes that appear to be particularly important protective factors against adolescent SUDs are monitoring and supervision [12] and communication with the adolescent [13]. Parental monitoring has been identified as a pivotal factor in the initiation of adolescent substance use [10, 14–17] and several studies have implicated parental monitoring as a mechanism underlying adolescent substance use. For instance, one study of 4731 adolescents found that perceived parental monitoring had unique mediating effects on adolescent substance use [18], while other studies concluded that lax parental monitoring was one of the pathways through which parental substance use influenced adolescent substance use [19, 20]. Parental communication also appears to have important protective influences for adolescents with SUDs [10, 21, 22], with studies suggesting that the content, context, style and timing of communication about substance use are all important deterrents of use [23]. Several studies suggest that parenting interventions should target parent-teen communication about the parents’ attitudes toward substance use [24, 25]. One study [26] found that communicating strong parental norms against teen substance use reduced the risk of initiation in early adolescence, while two studies found that communicating disapproval of substance use predicted less alcohol use later in adolescence [24, 27].

Systematic reviews and meta-analyses of treatment approaches for adolescents with SUDs [28–31], including one conducted by this protocol’s Principal Investigator (PI; protocol first author) [32], have found that intervention models that include parents outperform other approaches and are supported by high quality evidence. In Kumpfer et al.’s [33] review of interventions for adolescents with SUDs, family-based interventions that involved a parent or caregiver had average effect sizes 2–9 times larger than adolescent-only interventions. A more recent meta-analysis by Tanner-Smith and colleagues [31] similarly found that family-based interventions outperformed other evidence-based behavioral treatment approaches. Numerous other studies have provided evidence that parent involvement in treatment can improve adolescent engagement, retention, and substance use outcomes [34–36]. This body of research provides justification for: (1) working with the parents of adolescents in residential treatment for SUDs and (2) for targeting parenting skills as a potential mechanism of change in adolescent SUD treatment outcome.

### Limitations of prior parenting interventions

A key limitation of existing interventions for parents of adolescents with SUDs is the difficulty ensuring that parents receive an adequate “dose” of the intervention. In a 2014 systematic review of adolescent SUD interventions [28], those family models identified as “well-established” or “probably efficacious” ranged in intensity from 12 to 24 in-person sessions. Such a high dose of in-person sessions may be especially challenging for parents of adolescents transitioning out of residential SUD treatment, who are often difficult to engage in follow-up care [5]. Studies of adolescents discharged from residential SUD treatment demonstrate that only 36% will attend any continuing care sessions in the community [37]. Moreover, data from community treatment samples indicate that most adolescents who do attend treatment will drop out prematurely [38]. The few studies that have reported high retention rates of parents (i.e., [5, 8]; average of 14.4 sessions of continuing care) have predominantly relied upon home-based sessions or visits, an approach that may be challenging to implement within community programs that often lack adequate staffing and financial resources [39]. To date, there are no treatment models designed specifically to enhance parental engagement or increase parenting skills following the adolescent’s discharge from residential treatment. Thus, the current protocol addresses gaps in extant treatment options by adapting a low-cost, low intensity treatment model that addresses parent engagement and targets key parenting processes (i.e., monitoring and communication) within the prototypic constraints of an acute community residential program.

### Rationale for technology-assisted interventions

To improve upon currently available parenting interventions, it is critically important that any new approach is not only effective, but also acceptable and feasible for both residential treatment center staff and parents. Technology-assisted interventions can potentially better fit the needs of both clinical staff and parents in a number of ways [40, 41]. First, technology-assisted programs have the key advantage of balancing flexibility with fidelity, by allowing the content to be individually tailored to the patient’s specific needs, while retaining core content elements. Second, technology-assisted interventions can be delivered at low-cost with low burden on staff time, thereby ameliorating well-documented challenges to implementing evidence-based interventions such as limited financial resources, front line staff availability, and supervisory support [42, 43]. Third, online programs delivered via computer or smartphone are especially promising due to ease of access. Eighty-seven percent of American adults report using the internet, including 77% of adults in households that earn below $30,000 [44]. Rates of internet usage among adults age 30–49 (a common age range of parents of adolescents) are even higher at 91% [44]. Fourth, recent studies have provided promising support for the effectiveness of computerized or online treatments for adolescents with substance-related problems as additives or alternatives to clinic-based therapy [45–49]. However, these studies have primarily focused on cigarette use [50], prevention rather than intervention [51, 52], and on the adolescent only without involving parents [53].

One online parenting intervention that has promise for parents of adolescents in residential SUD treatment is Parenting Wisely (PW [54]). PW is an internet-based, self-paced program that consists of nine modules. Each module addresses how to handle a common parenting problem including: addressing adolescent substance misuse, monitoring friends, and improving parent–child communication. Five prior studies (two open trials [53, 55], one control-matched trial [56], and two small randomized controlled trials [57, 58]) have specifically tested PW as an intervention for parents of children with behavior problems. Findings across these studies indicated that parental receipt of PW is associated with improvements in parental knowledge and social problem solving skills and reductions in child problem behaviors (measured by the Eyberg Child Behavior Inventory [59]), with medium to large effect sizes.

A recent NIDA funded pilot study (R44 DA026658) was the first to evaluate PW as an adjunct intervention among parents of adolescents with SUDs, all of whom were court referred due to marijuana use. The study compared an educational substance use intervention to a motivational enhancement therapy (MET) with PW. Unpublished pilot analyses of the 3-month outcomes (Don Gordon, private communication) based on 128 cases demonstrated that MET + PW had statistically significant beneficial effects relative to education on parent reports of adolescent emotional problems, conduct problems and total behavior problems. In addition, MET + PW produced reductions in adolescent alcohol and drug use relative to education, but the difference was not statistically significant. Further analyses indicated a significant dosage effect for PW: completion of more PW modules was associated with significantly greater reductions in adolescent alcohol and drug use. Effect sizes were generally small in magnitude. This body of work provides evidence in support of PW as a means of reducing child behavior problems [36–38], with some pilot data suggesting that PW has the potential to address adolescent SUDs.

Although the data in support of PW are promising, use of the program is limited when delivered online only. For instance, a 2013 study [60] compared four different PW delivery models and found that delivery of PW content via 5 weeks of in-person groups resulted in significantly larger improvements in parenting skills than PW delivery via internet only. This is consistent with data indicating that new technology-delivered applications are often limited by lack of sustained use; for instance, in 2015, 25% of phone apps that were downloaded were only used once in the first 6 months of ownership [61]. The true challenge therefore is not developing new and exciting technology-delivered interventions, but the “need for collaborative techniques to enhance and maintain usage in vivo to improve therapy outcomes” [62]. A number of studies of adults and adolescents have shown that technology-delivered interventions have positive, sustained effects when combined with low intensity in-person support [63–65]. Thus, instead of comparing in-person versus online delivery, this protocol tests the potential value of an *additive* (in person + online) technology-assisted approach. Furthermore, this protocol augments the PW program with other technology-assisted parental engagement strategies.

### Overview of adaptations to Parenting Wisely tested in this protocol

This protocol examines three adaptations to the PW online model based on qualitative pilot data from 13 parents (7 parents of females, 6 parents of males), 11 adolescents (5 females, 6 males) in residential treatment, and three residential treatment staff (see Table 1 for summary of themes) that were collected as part of a larger qualitative study [66, 67]. First, based on qualitative data indicating that parents did not want to lose the “human element” of treatment, we offer parents up to four in-person coaching sessions. The first two sessions are offered while the adolescent is receiving residential treatment, the time parents described as easiest to attend sessions, in order to capitalize upon parental involvement with the residential treatment program. Second, in response to feedback that parents wanted convenient access to an expert and to other parents post-discharge, parents will receive access to an expert-facilitated online message board. Finally, in order to reinforce usage of PW and the online parent board, parents will receive two text messages weekly containing links to videos of parenting skills, messages of encouragement, and links to the online message board. Such supports are designed to sustain parental engagement, reinforce important parenting skills, and provide easier access to a treatment provider during a time when adolescents are at high risk of negative outcomes, but parents reported it was especially difficult to attend in-person sessions.

**Table 1:** Qualitative themes about parenting interventions revealed in pilot data collection with 13 parents, 11 adolescents, and 3 residential staff

Theme	Group	Findings
Parent only sessions	Parent	Parents wanted more (and not fewer) “parent only” sessions
		Parents wanted “parent only” sessions incorporated into existing visits/obligations when possible
	Staff	Residential staff described typical “parent only” sessions as focused on case management
Parenting skills	Parent	Parents were interested in learning new ways to manage their teen’s behavior
		When asked to rate their interest in receiving a parenting skills intervention, all but one parent gave the highest possible rating (very interested)
	Adolescents	Adolescents thought their parents would benefit from skills in the areas of stress management, communication, and conflict reduction
	Staff	Residential staff felt that parents most needed help with monitoring and communication
Timing	Parents	Parents described a mismatch between when it was easiest to attend sessions (while the teen was in residential) and when sessions were typically offered (immediately post-discharge)
		Parents stated a preference for fewer in-person sessions post-discharge
	Staff	Residential staff estimated that at least 2/3 of parents attended sessions while the teen was in treatment, but that fewer than 1/3 attended post-discharge sessions
Computer	Parents	Parents unanimously expressed comfort using computers or smartphones
		Virtually all parents reported looking for information about their teen’s treatment via the internet
		Multiple parents said they wished they could connect with an expert and other parents online
Delivery	Parents	Parents liked the idea of technology as an add-on but didn’t want to lose the “human element”
		Parents rank ordered different delivery options for a parenting intervention: parents most preferred mixed (in-person + computer) support and least preferred computer only
	Staff	Residential staff had a strong preference for a mixed (in-person + computer) approach over computer only

### Study objectives and specific aims

The overarching goal of this protocol is to evaluate the feasibility, acceptability, and preliminary effectiveness of the adapted PW model (PW+) among parents of adolescents (age 12–17) preparing for discharge from acute residential SUD treatment. The project has two phases. In Phase 1, a pilot open trial tests the PW+ protocol with 10 parent-adolescent dyads. Feedback from residential staff, parents, and adolescents will be used to: (a) finalize a manual for adjunct individual coaching sessions, (b) develop coach training materials, and (c) refine the delivery and content of technology-assisted engagement strategies. During Phase 2, a pilot randomized controlled trial with 60 parent-adolescent dyads will evaluate the effectiveness of the PW+ intervention as an adjunct to residential standard care (SC). Combined, the activities in these two study phases will address three Specific Aims.

#### Specific aim 1

To examine the feasibility and acceptability (primary protocol outcomes) of the PW+ technology-assisted intervention among parents of adolescents in residential SUD treatment.

#### Specific aim 2

To examine the preliminary effectiveness of the PW+ technology-assisted protocol on adolescent substance use and associated high-risk behaviors (secondary protocol outcomes) over the first 24 weeks post-discharge.

#### Specific aim 3

To examine the preliminary effects of the PW+ technology-assisted on parental monitoring and communication (putative mediators of the intervention) over the first 24 weeks post-discharge.

## Methods

### Timing of key protocol elements

Table 2 depicts the timing of key protocol elements including enrollment, allocation (in the randomized trial), intervention, and assessments. Two issues related to the timing of protocol components bear mention. First, key procedures (i.e., consent, baseline assessment, randomization, intervention) are conducted immediately after the adolescent’s admission to residential treatment. We start the protocol immediately instead of waiting until after discharge, due to qualitative pilot data (see Table 1) indicating that parents viewed the post-discharge period as overwhelming, and that parents had concerns about their ability to schedule face-to-face sessions during this period. Starting immediately after admission also capitalizes upon a critical intervention window while the teen is in residential treatment. Second, the open trial has one follow-up assessment at 6-weeks post-discharge that focuses on feasibility and acceptability, while the pilot randomized trial has a longer follow-up assessment schedule of 6-, 12-, and 24-weeks post-discharge focused on preliminary effectiveness. Prior studies by members of our team [68, 69], have suggested that brief interventions have their strongest effect between baseline and 24 week follow-up, with significantly less change over subsequent assessments. Having three follow-up assessments during the first 24 weeks post-discharge will serve to elucidate the change that is likely to occur immediately following treatment.

**Table 2:** Timing of enrollment, intervention, and assessment activities

Timepoint	Enrollment	Allocation	Residential treatment		Post-discharge period		
	−t1	0	~2 week stay	Discharge	+6 weeks	+12 weeks	+24 weeks
*Enrollment*							
Informed consent/assent	X						
Allocation		X					
*Intervention*							
Standard care			X	X	(Optional)		
PW+ elements							
Coaching (up to 4 sessions)			X	X	X		
Computer program			X	X	X	X	
Text messages					X	X	
Parent online message board					X	X	
*Assessment*							
Primary outcomes: feasibility and acceptability			X	X	X	X	
Secondary outcomes: adolescent outcomes	X				X	X	X
Putative mediators	X				X	X	X

### Residential center

Evaluation of the PW+ intervention will occur at the only acute residential community SUD treatment program in Rhode Island. The facility contains 16 beds and is located about 20 min outside the state capital in a suburban area. It recently affiliated with the local hospital, but continues to operate as an independent community-based program. Average length of stay is 6–10 days. About five adolescents are admitted per week on average, which equates to approximately 260 admissions per year. Based on past year census data, about 30% of adolescents at the residential treatment center are racial/ethnic minorities, with Hispanic (about 20%) and African-American (about 10%) being the most commonly represented minority groups. There is approximately even representation of male and female adolescents. In the pilot qualitative sample, median household income was $30,000, with an extremely wide range from $18,000 to over $200,000.

### Participants

A total of 70 parent-adolescent dyads (10 in Phase 1/open trial and 60 in Phase 2/pilot randomized trial) will be recruited from the acute residential treatment center. To qualify for the study, parents must meet these inclusion criteria: (1) be the parent or legal guardian of an adolescent, aged 12–17 years; (2) had adolescent admitted to residential treatment due to problems related to substance use; (3) will be the primary guardian living with the adolescent immediately post-discharge; (4) willing to receive a parenting intervention; (5) fluent in English or Spanish; and (6) willing to provide written consent and adolescent willing to provide written assent. Adolescents automatically qualify if their parents meet these criteria. The only exclusion criteria are conditions that might preclude the adolescent’s participation in a 2–3 h interview (e.g., mania, psychosis, and cognitive impairment). Based on recent enrollment data, we conservatively expect at least 50% of new admissions to be eligible (with discharge to another facility, discharge to another caregiver, or parent unwilling to receive an intervention as primary reasons for exclusion). To meet our recruitment goal of 70 parent-adolescent dyads, we will need to enroll 25–30% of eligible parents over the first 2 years of the study.

### Study enrollment and randomization

In accordance with institutional review board procedures at Brown University and Rhode Island Hospital, parents of all newly admitted adolescents will receive a Consent to Contact (CoC) form as part of the standard intake process at the residential center. Parents that sign a CoC form will then be contacted by research staff in one of two ways. If the adolescent is admitted Monday through Friday during normal business hours (which accounts for about 80% of new intakes), research staff located on site will immediately approach the parents and invite them to initiate the consent process. If the adolescent is admitted after hours or on weekends, research staff will contact the parents using the preferred contact information provided.

Parents will provide written informed consent and adolescents will provide written assent in separate private rooms at the residential center. If an adolescent chooses not to participate, the parent will be given general feedback that the family did not qualify so as to protect the adolescent’s privacy. Parents and adolescents may choose to conduct the informed consent process and all other study procedures in English or Spanish.

Parents will be given the choice whether to return for the baseline assessment or complete it immediately following informed consent. In the Phase 2 trial, randomization will occur within 24 h after the baseline assessment. Parent-adolescent dyads will be randomized to either SC or PW+ using urn randomization balanced on sex, severity of substance use, and preferred language (English vs. Spanish). Randomization will be conducted by a Co-Investigator, not involved with the assessment process, as soon as a parent consents to participate. Condition assignments will be placed in sealed envelopes and given to the independent evaluator. The evaluator will give parents their sealed envelope indicating that their treatment condition immediately after completion of the baseline assessment. If the parent is assigned to PW+, the evaluator will schedule the first in-person coaching session.

### Therapist training, fidelity, and competence

Treatment will be delivered by two parent coaches. The coaches are part-time BA-level research staff, one of whom is bilingual in English and Spanish. The decision to use research staff as parent coaches was made in consultation with leaders of the residential facility, who wanted indication that the PW+ intervention was viewed favorably by parents before allocating substantial staff effort to the program. We specifically agreed to hire BA-level research staff with no clinical experience since these credentials are lower than those of program staff. Each coach will deliver both PW+ and SC sessions to prevent confounding of therapist effects. The study PI will train the two coaches. Parent coaches will review the PW computerized program and the manual for in-person sessions. Acting as a standardized patient, a study Co-Investigator with extensive experience treating adolescents with SUDs will role-play the four coaching sessions with each coach. These role plays will be recorded. The study PI will view these role plays, complete measures of therapist adherence and competence, and provide feedback to each counselor. Competency will be rated with six items that comprise the “General Therapeutic Skills” section of the Cognitive Therapy Rating Scale (CTRS) [70, 71]. A score >24 is the criterion to indicate competence. A 12-item adherence checklist for each coaching session has been developed. The coaches will be required to earn a satisfactory consensus CTRS score of >24 on three of four role plays and satisfactory adherence ratings (9 out of 12 elements) to be assigned an actual participant. Once the coaches are assigned participants, the PI will review and rate the first two tapes for adherence and competence. If ratings are satisfactory, the PI and a study Co-Investigator will collectively review 25% of tapes moving forward. The team will also rate 25% of SC sessions to ensure there is not drift between PW+ and SC sessions. The PI will meet weekly with the coaches for supervision focused on how to deliver PW+ with adherence, while flexibly tailoring content to each parent’s unique presenting concerns.

### Standard care (SC)

SC consists of the standard treatment elements offered to all residential patients at the partner residential center. Adolescents receive about 25 h of treatment per week. Before an adolescent is discharged (typical length of stay 6–10 days), parents are typically offered two family therapy sessions (which typically use a family systems approach and include case management planning). Adolescents receive four to 5 h of individual and group therapy sessions per day, consisting of psychoeducation and general skills building. Medication management is offered by a psychiatrist as needed. Prior to discharge, parents also receive standard discharge planning, which consists of either a referral to a new outpatient therapy provider or return to a prior outpatient therapy provider. As indicated, adolescents also receive referrals to a psychiatrist or local self-help groups. Post discharge, families are offered up to two check-in sessions to review the adolescent’s progress and troubleshoot any difficulties complying with the treatment plan.

### Adapted PW program

In addition to the SC elements, parents in the PW+ condition will receive the following elements: PW online modules, in-person coaching sessions, personalized texting, and an expert-moderated online parent board. All study materials will be translated into Spanish by a bilingual English and Spanish research staff member, and reviewed for accuracy by a Study Co-Investigator. Parents will be able to choose whether they prefer to receive materials in English or Spanish.

#### PW online modules

PW (Parenting Wisely/Ser Padres Con Sabiduría) is a self-administered, interactive, multimedia online program. PW contains video vignettes of ten common family problems (e.g., adolescent substance abuse, curfews, household chores, monitoring of friends, sibling conflict, improving in school, getting up on time, sharing technology, etc.). For each problem, parents progress through three activities: viewing of a video clip of a family struggling with the problem; selection of one of three possible solutions to the problem; and receipt of feedback about the solution selected. Feedback is presented via a video enactment of the selected solution and an explanation of the solution’s pros and cons. The goals of the feedback are to explain why ineffective solutions lead to problems and discuss how effective parenting solutions can prevent and resolve common family issues. Each vignette emphasizes six core behaviors: (1) problem solving; (2) limit setting; (3) praising; (4) effective communication through “I messages” and active listening; (5) supervision and monitoring, and (6) clear communication of adolescent’s behavioral expectations. Parents receive a parent workbook outlining all 10 vignettes, along with the potential problems and solutions. The workbook also contains a glossary of terms, sample behavior charts, and practice exercises. Parents create a unique login upon enrollment and can complete sessions at their own pace. Depending on the user’s speed and depth of use, the PW program typically takes between 3 and 5 h to complete.

#### Individual coaching sessions

Individual coaching sessions are designed to individually tailor the PW skills to each parent’s presenting concerns. The in-person coaching sessions correspond with four of the PW vignettes: substance use, limit setting, parental monitoring, and parental communication. The initial session is 60–75 min and consists of three sections: (1) rationale for parenting skills, (2) review of PW substance use module (in session), and (3) practice applying the skill to a current problem. Subsequent sessions follow the same outline and last 45–60 min, with active use of the PW program comprising about half the session. If parents introduce problems/complaints about their adolescent, the coach will first provide validation and then discuss how to apply a pertinent PW skill. Following discharge, the coach will also direct the parent to use the online parent board for additional support.

#### Text-based messaging

Following discharge, parents will receive text messages twice weekly for 12 weeks. The texts are intended to: (a) reinforce PW skills, (b) motivate continued PW usage, and (c) encourage participation in the online parent message board. In recognition of the ubiquity of text messaging in the general population [44] as well as in health-related research and clinical care [72], the PW developer has developed a set of text messages and videos to be delivered in conjunction with PW. In the open trial (Phase 1), it is possible that we will refine the text-messaging protocol (e.g., content, format, and frequency) based on feedback from parents during the exit interviews (described further below). One of the weekly messages will reinforce material taught in PW (e.g., a reminder statement about a parent strategy such as monitoring or communication) along with a link to a video clip enacting a core PW skill.

The second weekly text message will be encouragement for the parent to either practice PW vignettes or participate in the online parent message board. This protocol intentionally does not use interactive texting; instead, text messages will direct parents to ask questions via the parent board, an approach that we believe will ultimately be more transportable. Nonetheless, this limited application of texting is a potentially important ingredient to extending parents’ engagement in the parenting skills intervention, similar to the use of texting to provide support for smoking cessation [73] and to promote uptake of flu vaccinations [74] and outpatient clinic attendance [75].

#### Online parent board

Parents will be given access to a secure, expert-moderated website containing basic substance use and parenting skills information, links to sections of PW, as well as an interactive message board. Parents will be able to submit questions anonymously using the same unique login as for the PW program. All submitted questions will be reviewed by the project team to determine suitability for the board. Questions posted on the message board will be answered by the research team within 48 h. At the start of the treatment program, parents will be informed that the purpose of the parent online message board is to ask parenting related questions and not to request crisis support; requests for crisis support will be handled by the PI, a licensed clinical psychologist, and will not be shared on the message board. Answers to questions posted on the message board will direct parents to a specific PW module(s) most relevant to their parenting question. Participation on the message board will be encouraged for 12 weeks post-discharge, with the link to the board continually reinforced through text messages. The online message board will use the same login and password as the PW website, and the online message board will continually provide links to the PW website, to promote consistent use across platforms.

### Exit interviews

During the open trial phase, parents will complete exit interviews about their impressions of the PW+ program after the 6-week follow-up. Parent exit interviews contain questions about each aspect of the intervention, including the in-person sessions (e.g., number, content, format), text messages (e.g., content, frequency, format), and online message board (e.g., ease of access, content, format). Parents will also complete the 16-item Consumer Satisfaction Questionnaire (CSQ; [76]), which rates their satisfaction with treatment delivery, their adolescent’s treatment progress, and their ability to manage their adolescent’s problems. Parents rate 11 items along a 7-point scale (α = .91) and answer five open-ended questions about their experience in treatment. If more than one parent expresses the same concern with a protocol element (e.g., the frequency/number of in-person sessions, the frequency/content of texts, an aspect of the message board), then the element will be flagged for possible modification by the research team.

### Assessments

Parents and adolescents will complete a baseline assessment in-person shortly after their admission to residential treatment. Adolescents will be assured of confidentiality and a National Institute of Health Certificate of Confidentiality (CoC) will be obtained, as adolescent self-report use has shown reliability when confidentiality is assured [77–80]. The CoC will allow the research team to refuse to disclose names or other identifying characteristics of research participants in response to legal demands such as subpoena of records. Follow-up assessments will be conducted by an independent evaluator who is blind to condition. During the open trial, there will be follow-up assessment at 6 weeks post-discharge, while during the pilot randomized trial there will be three follow-up assessments at 6, 12, and 24 weeks post-discharge. Follow-up assessments will be scheduled in-person at the residential treatment center; if a parent no-shows more than twice for a follow-up assessment, then research staff will attempt to conduct the follow-up sessions at a location of the family’s choosing (i.e., home, school). The baseline assessment will be 1.5–2 h and the follow-up assessments will be about 1.5 h (see Table 3 for overview of measures).

**Table 3:** Overview of protocol measures

Construct	Measurement/scale	Data obtained
Feasibility	Participation rates	Percent of parents who participate in the study
	Parent engagement	Number of coaching sessions attended, online usage of PW and parent message board (i.e., logins, sessions completed, page views, time spent), number of texts viewed
Acceptability	Withdrawal rates	Percent of parents who withdraw from study
	Exit interviews (open trial only)	Open-ended questions on perceptions of in-person sessions, text messages, computer program, and online message board
	CSQ	Satisfaction with treatment delivery and ability to manage their adolescent’s problems
Adolescent substance use	GAIN days of use	Days of alcohol use, marijuana use, and other drug use
	GAIN SFS	Averages percent of past 90 days during which there was use of alcohol, marijuana, or heavy drugs; intoxication; and failure to perform activities due to use
	Gain SUDS	Assesses substance use disorder symptoms in line with both DSM-IV and DSM-V criteria
	Biological markers	Saliva alcohol screens (12 h window) and 8 panel urine drug screens testing for marijuana, MDMA, cocaine, amphetamines, methamphetamines, opiates, oxycodone, and benzodiazepines
Adolescent high-risk behavior	GAIN risky sex	Series of items about risky sexual behavior over the past 90 days including number of partner, number of sexual contacts, and number of times had unprotected sex
	GAIN days legal	Days of legal involvement over the past 90 days
	GAIN days school	Days of school attendance/truancy over past 90 days
Parenting	PMQ	Parental monitoring and sources of parental monitoring
	PAC	Assesses positive and negative aspects of general parent-teen communication
	FAsTask video code	In vivo problem solving tasks focused on: limit setting; substance use norms; monitoring and listening

*CSQ* customer satisfaction questionnaire, *GAIN* global appraisal of individual needs, *SFS* substance frequency scale, *SUDS* substance use disorder scale, *PMQ* parental monitoring scale, *PAC* parent-adolescent communication scale

We selected adolescent measures to be as consistent with SC as possible. The residential center typically obtains a urine screen and administers a comprehensive adolescent interview to assess substance use and functioning across multiple life domains. We therefore combine biological markers with the Global Appraisal of Individual Needs—Quick (GAIN-Q3 [81]), a well-validated structured interview that assesses adolescents’ problems across several domains: substance use, school, mental health, physical health, risk behaviors, work, and sources of stress. The GAIN-Q3 substance use scales used in this protocol have demonstrated sound psychometric properties including internal consistency [82], high concurrence with the Timeline Followback Method [83], ability to predict SUD diagnoses made by blind raters (κ = .91) [84], and ability to predict substance-related problems as well or better than biometric markers [82]. The PI is a certified trainer of the GAIN family of instruments with experience analyzing GAIN data [85–88] and will train the independent evaluators.

To explore potential mediators of change, we augment the GAIN-Q3 with two brief measures of parental monitoring [89] and communication [90] (completed by both the parent and adolescent), as well as an in vivo parent-adolescent interaction task [91, 92]. The self-report measures have demonstrated good internal consistency and have correlated well with adolescent involvement in risky behavior, adolescent maladjustment, and family discord [89, 90], while the in vivo task has shown high inter-rater reliability [69]. These parenting measures will only be completed by those parents with have primary custody of their adolescent. If a parent loses custody post-discharge, parenting measures will not be administered (treating parent data as missing).

### Retention procedures

Multiple steps will be taken to prevent attrition from the study follow-up assessments. First, during the baseline assessment, multiple sources of contact information will be recorded for parents and adolescents. In addition to extensive personal contact information, parents will provide detailed contact information of two friends or family members (“locators”) who can be contacted if research staff is unable to reach them. Follow-up reminders will be made via phone, text, and/or mail, depending on the parents’ preferences. The research team will meet weekly to review no-shows and outreach attempts, and discuss plans to connect with missing participants. Parents and adolescents will each receive an escalating schedule of compensation in the form of gift cards for completing the baseline ($40) and three follow-up assessments ($50, $50, $60, respectively).

### Data analysis plan

All 60 parents will be included in statistical analyses consistent with an intent-to-treat (ITT) model. Prior to the main analyses, descriptive statistics will be run on key variables to examine distributional properties, identify outliers, and transform variables as needed. The two treatment conditions will be compared on baseline demographic and clinical variables using basic two-group independent sample t-tests. Using a *p* < .05 criterion, significant pre-existing baseline differences between conditions on any variables will be controlled in subsequent analyses.

GAIN-Q3 data will be entered into the GAIN’s proprietary software, which offers instant error checks and validation reports to prevent missing data. Other forms will be entered directly into study laptops using REDCap (Research Electronic Data Capture), a secure web-based application, to reduce missing data and entry errors. Attrition analyses will compare follow-up completers and non-completers on baseline clinical and demographic variables to determine if they differ systematically. If no systematic differences are found between completers and non-completers, then missing data will be estimated using multiple imputation methods with five datasets, an approach that has been shown to generate unbiased estimates when data are missing at random or missing completely at random [93].

#### Primary outcomes: feasibility and acceptability

In the open trial and randomized controlled pilot trial, feasibility will be examined via enrollment rates (>80%) and indicators of parental engagement, with the following preliminary targets: attendance of >1 coaching sessions pre-discharge and >1 sessions post-discharge for >75% of parents, and online usage patterns of 2+ PW logins, 2+ parent board logins, and 1+ h logged in for >75% of parents. Criteria for acceptability include withdrawal rates (<20%), satisfaction ratings (>80% of items on the CSQ rated “agree” or “strongly agree”) and positive feedback from exit interviews with parents and therapists. Criteria will be finalized based on parent and staff feedback in Phase 1.

#### Secondary outcomes: adolescent variables

Secondary outcomes include adolescent substance use and associated high risk behaviors (i.e., sexual risk-behavior, truancy, and criminal involvement). Analyses will compare trajectories of use over the post-discharge period, controlling for baseline status as a covariate. For each dependent variable, separate repeated measures Analysis of Covariance will test the influence of condition, controlling for the following covariates: baseline status on the dependent variable, baseline differences between groups, and any mental health or substance use treatment received over the follow-up period. The effect of PW dosage on each dependent variable will be calculated and partial eta squared (η2) will be used to estimate the proportion of variance caused by PW. The primary analytical goal will be to provide measures of association and 95% confidence intervals to reveal the effect size of PW on each dependent variable.

#### Putative mediators: parental communication and monitoring

The study will be underpowered to test mediation, but will examine the effects of PW on putative mediators. A repeated measures Analysis of Covariance will whether assignment to the PW+ condition has a significant effect on either of the putative mediators, controlling for any treatment received. In addition, we will calculate change in the putative mediators over each time interval as well as change in the dependent variables over each time interval. Correlations between change in the putative mediators and change in the dependent variables will be examined; significant positive correlations greater than *r* = .20 will be flagged as worthy of further analysis in a future trial. The goal of these analyses is to find promising trends to pursue in a larger fully powered trial.

For both the secondary outcome analyses and the mediator analyses, the investigators will also attempt to fit latent growth models to the data. Latent growth models represent a flexible approach to analyzing change that enable a comparison of conditions at each time point while accounting for the correlation of observations over time [94]. Although convergence is challenged in a small sample, latent growth models have been fit to samples as small as *n* = 22 [95, 96]. The study PI has conducted multiple analyses using latent growth models [32, 97, 98] and will lead the longitudinal analyses.

#### Sample size and power

The study will recruit 60 participants for the randomized trial. Estimating that 15–20% may be lost to follow-up, there will likely be 50 participants with complete parent-adolescent data. We will use these participants to make judgments regarding feasibility and acceptability. Effect size estimates in prior PW studies have ranged from small to very large; the only study conducted among adolescent substance users found small to medium effects, but unlike this application, that study did not offer in-person support or an online parent board (supports that may contribute to larger effect sizes). Furthermore, even small effects, if obtained by delivering content electronically via a highly scalable platform that can reach large numbers of parents, can have significant public health implications. Despite issues with effect size stability in small samples [99], a small to medium effect paired with high levels of feasibility and acceptability would therefore be an indication that the adapted PW intervention should be tested in a larger trial. Based on the detected effect size, post hoc power analyses will be conducted to determine the number of parents required for a fully powered trial. For instance, for power of .8, a small effect size (d = .3) would require a sample of 175 per group, while a medium effect size (d = .5) would require a much smaller sample of 64 per group.

## Discussion

This protocol evaluates whether a technology-assisted parenting intervention can improve the treatment outcomes of adolescents discharged from acute residential treatment. The approach builds upon qualitative data collection with parents of adolescents in residential treatment and combines both in-person and technology-delivered intervention elements to improve parental engagement at a time when parents have especially poor attendance.

Findings from this protocol will be subject to important limitations. First, the residential program is based in Rhode Island about 15 min from a major urban center. Results of this protocol may not generalize to programs in other states or more rural regions. Second, the PW+ condition and the SC condition do not offer an equivalent dose of contact time. It is therefore possible any favorable results associated with PW+ may be accounted for by a higher “overall” dose of treatment (e.g., coaching sessions, text messages, time spent on the parent message board, time spent completing the computer program). Because this is a treatment development protocol, we focus our resources on developing and pilot testing the experimental condition. If results are promising, we will attempt to control for contact time in a fully powered trial.

By adapting and evaluating an intervention for parents of adolescents in residential treatment, this protocol has the potential to advance treatment by: (1) addressing a high-needs population; (2) targeting parenting skills (putative mediators) that have been shown to influence adolescent substance use and related risk behaviors; (3) working with parents at a critical treatment juncture (i.e., immediately prior to and following the adolescent’s discharge from residential treatment); and (4) developing a model with potential for widespread dissemination in community residential programs. If results of this protocol are promising, future research will attempt to evaluate the PW+ protocol in a fully powered protocol. To accelerate the translation of research to practice, the fully powered trial will use a type 1 hybrid effectiveness-implementation design that simultaneously gathers data on the effectiveness of the model under real-world conditions and collects data on barriers and facilitators to effective implementation [100].

## Abbreviations

CSQ: consumer satisfaction questionnaire
CTRS: Cognitive Therapy Rating Scale
GAIN-Q3: global appraisal of individual needs—quick version
PI: principal investigator
PW: Parenting Wisely
SC: standard care
SUD: substance use disorder
